# Novel therapeutic targets in salivary duct carcinoma uncovered by comprehensive molecular profiling

**DOI:** 10.1002/cam4.2602

**Published:** 2019-10-14

**Authors:** Stacey M. Gargano, Wijendra Senarathne, Rebecca Feldman, Elena Florento, Phillip Stafford, Jeffrey Swensen, Semir Vranic, Zoran Gatalica

**Affiliations:** ^1^ Department of Pathology, Anatomy and Cell Biology Thomas Jefferson University Hospital Philadelphia PA USA; ^2^ Caris Life Sciences Phoenix AZ USA; ^3^ College of Medicine QU Health Qatar University Doha Qatar

**Keywords:** biomarkers, head and neck cancer, molecular genetics, next generation sequencing

## Abstract

Salivary duct carcinoma (SDC) is a rare, aggressive salivary gland malignancy, which often presents at an advanced stage. A proportion of SDC are characterized by *HER2* amplification and/or overexpression of androgen receptor (AR), which could be targeted in a subset of patients, but the presence of AR splice variant‐7 (AR‐V7) in some SDC cases could result in resistance to anti‐androgen therapy. We evaluated a cohort of 28 cases of SDC for potentially targetable biomarkers and pathways using immunohistochemistry (IHC) and next‐generation sequencing (DNA and RNA) assays. Pathogenic genetic aberrations were found in all but 1 case and affected *TP53* (n = 19), *HRAS* (n = 7), *PIK3CA*, *ERBB2 (HER2)*, and *NF1* (n = 5 each); *KMT2C (MLL3)* and *PTEN* (n = 3 each); *BRAF* (p.V600E), *KDM5C* and *NOTCH1* (n = 2 each). Androgen receptor was expressed in all cases and 13 of 27 harbored the AR‐V7 splice variant (including a case without any other detectable genetic alteration). HER2 IHC was expressed in 11 of 28 cases. The majority of SDC cases had no biomarkers predictive of immunotherapy response: 5 cases exhibited low (1%‐8%) programmed death ligand 1 (PD‐L1) expression in tumor cells, 2 cases exhibited elevated TMB, and no samples exhibited microsatellite instability. Notably, the pre‐treatment biopsies from 2 patients with metastatic disease, who demonstrated clinical responses to anti‐androgen therapy, showed AR expression and no AR splice variants. We conclude that comprehensive molecular profiling of SDCs can guide the selection of patients for targeted therapies involving AR, HER2, PD‐L1, mitogen‐activated protein kinase, and PIK3CA pathways.

## Graphical abstract

Salivary duct carcinoma (SDC) is a genetically diverse malignancy associated with several potentially targetable molecular pathways that have been uncovered by comprehensive molecular profiling. Our study detected genetic aberrations in 27 of 28 cases of SDC, including targetable alterations involving PIK3CA/PTEN, BRAF, and HER2. In addition, androgen receptor splice variant AR‐V7 was found in approximately half of cases, which has implications in resistance to anti‐androgen therapy.

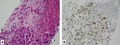

## 1 INTRODUCTION

Salivary duct carcinoma (SDC) is a rare, high‐grade salivary gland malignancy characterized by an apocrine phenotype and morphologic similarity to ductal carcinoma of the breast.^1^ Men over the age of 50 are most frequently affected, and the parotid gland is the most common site. SDC tends to present with an enlarging mass sometimes accompanied by facial nerve paralysis.^2^ With the exception of cases occurring as intracapsular SDC ex pleomorphic adenoma (PA), this tumor is highly aggressive. Patients often present with advanced stage disease including frequent nodal metastases, and overall survival is poor, with approximately 65% of patients dying from progressive disease within 48 months.^2^, ^3^ Standard management for SDC consists of surgical resection followed by radiation therapy; however, local recurrence and metastatic rates remain high, and treatment options for recurrent and metastatic SDC are limited.^3^ Therefore, there is an unmet need for targeted therapies to help better manage this aggressive malignancy.

Several characteristic findings in SDC have been exploited in the development of targeted therapy including the overexpression of HER2/*neu* (*ERBB2*) found in 37% of SDC *ERBB2* gene amplification is found in 72% of overexpressing cases.^2^ Clinical responses to HER2‐targeted agents, alone or in combination with chemotherapy, have been reported in individual patients with HER2 + SDC.^4^, ^5^, ^6^, ^7^ Additionally, androgen receptor (AR) expression which is a defining feature of SDC, is found in up to 98% of cases.^1^, ^8^ Some argue that AR‐negative SDC is virtually non‐existent, as many such cases are either AR‐positive after repeat immunohistochemistry (IHC) testing, or are subsequently reclassified as a different entity with morphologic similarities to SDC.^1^ As a result, androgen deprivation therapy (ADT) has emerged as another potential targeted therapeutic strategy for SDC patients, potentially even in the adjuvant setting, and has shown promising results in several small studies.^9^, ^10^, ^11^, ^12^, ^13^

Despite some headway in targeted therapy for SDC, neither anti‐HER2 nor anti‐androgen therapies have been standardized or clinically validated. In addition, mechanisms of resistance to HER2‐based treatments and ADT may hinder its clinical benefit in some patients.^14^, ^15^ For instance, alterations in phosphoinositide 3‐kinase (PI3K) signaling, either by PIK3CA mutation or PTEN loss, may be involved in resistance to HER2‐targeted therapy.^15^ Additionally, the discovery of constitutively active AR splice variants, notably AR splice variant‐7 (AR‐V7), which are implicated in resistance to ADT in prostate cancer, has led to the discovery of these variants in SDC specimens as well.^14^, ^16^, ^17^, ^18^ A small study conducted by Cappelletti et al^16^ suggests that the presence of AR‐V7 in SDC cells may likewise affect response to ADT, but this requires more extensive investigation.

The treatment of numerous solid and hematologic malignancies (eg non‐small cell lung carcinoma, bladder carcinoma, melanoma, classical Hodgkin lymphoma) has been markedly improved due to therapy with immune check point inhibitors against programmed death 1 and its ligand PD‐L1 (immuno‐oncology [I‐O] treatment). Predictors of a response to these inhibitors include the expression of PD‐L1 in cancer or tumor‐associated immune cells (IC), tumor mutational burden (TMB), microsatellite instability (MSI) status, and the presence of tumor infiltrating lymphocytes.^19^, ^20^, ^21^ While immunotherapy has not yet been clinically validated for the treatment of salivary gland malignancies including SDC, several trials are underway. A study of Cohen et al^22^ revealed a modest therapeutic benefit (12% response rate) of pembrolizumab in the patients with PD‐L1 positive (≥1% positive cancer or IC) SDC, and it is thought that a combination treatment with chemotherapy may improve the clinical response rates (NCT03360890).

In this study, we report the findings from comprehensive molecular profiling focusing on identification of potential novel molecular targets and I‐O biomarkers in a cohort of SDC.

## 2 MATERIALS AND METHODS

### 2.1 Patients and samples selection

Twelve cases of primary SDC from the Department of Pathology at Thomas Jefferson University Hospital and 16 cases of SDC (6 primary cases and 10 recurrent/metastatic SDC cases, including one matched primary and metastatic tumor from the same patient) from Caris Life Sciences met the following inclusion criteria: Confirmed diagnosis of SDC and availability of sufficient formalin‐fixed paraffin‐embedded tissue from the primary and/or recurrent/metastatic tumor for molecular assays. All cases were re‐reviewed by a board‐certified pathologist to confirm the diagnosis and select appropriate slides for molecular profiling. A case was considered as SDC ex PA if histologic evidence of a PA was present in the same specimen, or if there was a clinical history of PA occurring previously in the same site. The Institutional Review Board of the Thomas Jefferson University Hospital approved the study (IRB #18D.142).

### 2.2 Immunohistochemistry

Quantification of AR staining (SP107 clone; Ventana) was performed with positivity defined as strong nuclear staining in ≥10% of tumor cells. Detection of the splice variant AR‐V7 was assessed at protein level by IHC (EPR15656; Abcam) (16 cases from Caris) and at mRNA level by anchored multiplex PCR for targeted RNA sequencing (ArcherDX) (12 cases from Thomas Jefferson University Hospital).

Positive HER2 IHC was defined as 3+ (strong membranous staining) in ≥10% of tumor cells. IHC for CD274 (PD‐L1) was performed using either 28‐8 (Agilent) or SP142 (Ventana) clones, and PD‐L1 positivity was defined as membranous expression in ≥1% cancer cells (TC).^23^ Additionally, PD‐L1 expression on the IC was recorded. All IHC assays were run with both positive and negative controls using fully automated staining platforms (Ventana‐Roche and DAKO‐Agilent). The assays were conducted in a CLIA/CAP/ISO15189 certified clinical laboratory (Caris Life Sciences).^23^

### 2.3 Next‐generation sequencing

Next‐generation sequencing (NGS) was performed using two commercially available platforms: the Caris Life Sciences (n = 22) and Foundation Medicine (n = 6). The Caris panel utilizes SureSelect XT biotinylated RNA probes from Agilent, to capture DNA fragments from the exons of 592 genes. Sequencing is performed using NextSeq instruments from Illumina. The complete list of the tested genes is available here: http://www.carismolecularintelligence.com/solid_tumors_international.^24^ The Foundation Medicine NGS platform was reported previously.^25^ For variant classification, variants of genes that were pre‐determined for their cancer related and clinical significance were interpreted by board‐certified clinical molecular geneticists at Caris and categorized as pathogenic, presumed pathogenic, variant of unknown significance, presumed benign, or benign according to American College of Medical Genetics and Genomics standards.

The TMB was assessed by counting the number of nonsynonymous missense mutations excluding common germline variants. TMB was considered high if ≥10 mutations/megabase were detected. This threshold was calculated based on Caris Life Sciences' cohort of 148 salivary gland carcinomas using the 80th percentile cutoff value as recently suggested by Samstein et al.^26^

Microsatellite instability status was explored in 18 cases by analyzing the microsatellite loci of the genes on the Caris panel, as reported previously.^23^ Gene copy number variations were assessed in 22 cases by comparing the depth of NGS sequence reads on the Caris panel to reads from a diploid control. Genes harboring with ≥6 copies were considered amplified.^23^, ^27^

ArcherDx FusionPlex Assay (ArcherDX) was used to search for gene fusions. Fifty‐three gene targets were analyzed in 12 SDCs (the panel is available here: https://www.carismolecularintelligence.com/tumor-profiling-menu/mi-profile-usa-excluding-new-york).^28^

## 3 RESULTS

### 3.1 Clinical characteristics of the cohort

Table 1 summarizes the cohort's characteristics; clinical follow‐up information was available for 12 patients (from Thomas Jefferson University Hospital). In accordance with known trends, the majority of the patients were elderly men with tumor in the parotid gland, and 12/12 patients with available clinical follow‐up presented with nodal (specifically stage pN2b) metastatic disease. In the cohort from Caris Life Sciences, 8 patients were with metastatic lesions (involving liver, lung, brain, skin, bone, and lymph nodes), 2 with local recurrences involving the skin, and 6 with primary tumors; clinical follow‐up was not available for this group.

**Table 1:** Summary of clinicopathologic features including patient demographics, tumor characteristics, treatment, and follow‐up

Clinicopathologic feature	N (%)
Age, mean (range) (y)	66.25 (41‐91)
Sex	
Male	24/27 (89%)
Female	3/27 (11%)
Primary tumor site	
Parotid	24/27 (89%)
Submandibular	2/27 (7%)
Lip	1/27 (4%)
Source of neoplastic tissue	
Primary tumor, de novo SDC	13/28 (46%)
Primary tumor, SDC ex pleomorphic adenoma	5/28 (18%)
Recurrent/metastatic SDC	10/28 (36%)
pT stage	
1	3/12 (25%)
2	0/12 (0%)
3	2/12 (17%)
4	7/12 (58%)
pN stage	
0	0/12 (0%)
1	0/12 (0%)
2	12/12 (100%) (all stage pN2b)
Treatment	
Chemoradiotherapy (CRT)	4/12 (33%)
Radiotherapy	7/12 (58%)
Anti‐androgen therapy	2/12 (17%) (both combined with CRT)
No therapy (surveillance)	1/12 (8%)
Follow‐up, mean (range) (mo)	18.4 (4‐45)
Clinical outcome	
NED	6/12 (50%)
AWD	2/12 (17%) (1 DM + 1 LR)
DOD	1/12 (8%)
DOC	1/12 (8%)
LTF	2/12 (17%)

Abbreviations: AWD, alive with disease; DM, distant metastasis; DOC, died of another cause; DOD, died of disease; LR, locoregional recurrence; LTF, lost to follow‐up; NED, no evidence of disease; SDC, salivary duct carcinoma.

### 3.2 Treatment and clinical follow‐up

Following surgical resection, 7 of 12 received adjuvant radiation therapy, 4 of 12 received chemoradiotherapy, and 1 patient received no additional therapy.

Two patients (1 male, 1 female) with distant metastatic disease were treated with anti‐androgen therapy (one received bicalutamide and the other received enzalutamide), both in conjunction with chemoradiotherapy. One patient had lung metastases at the time of diagnosis, and she was alive with stable pulmonary metastatic disease at 42 months after initial diagnosis. The other patient demonstrated a total reduction in metastatic tumor burden of 28% after two scans, and then showed stable disease at the time of his third scan but passed away soon afterward. Neither of these patients had AR‐V7 detected in their tumor samples.

### 3.3 Molecular characteristics of SDCS

**Figure 1:**
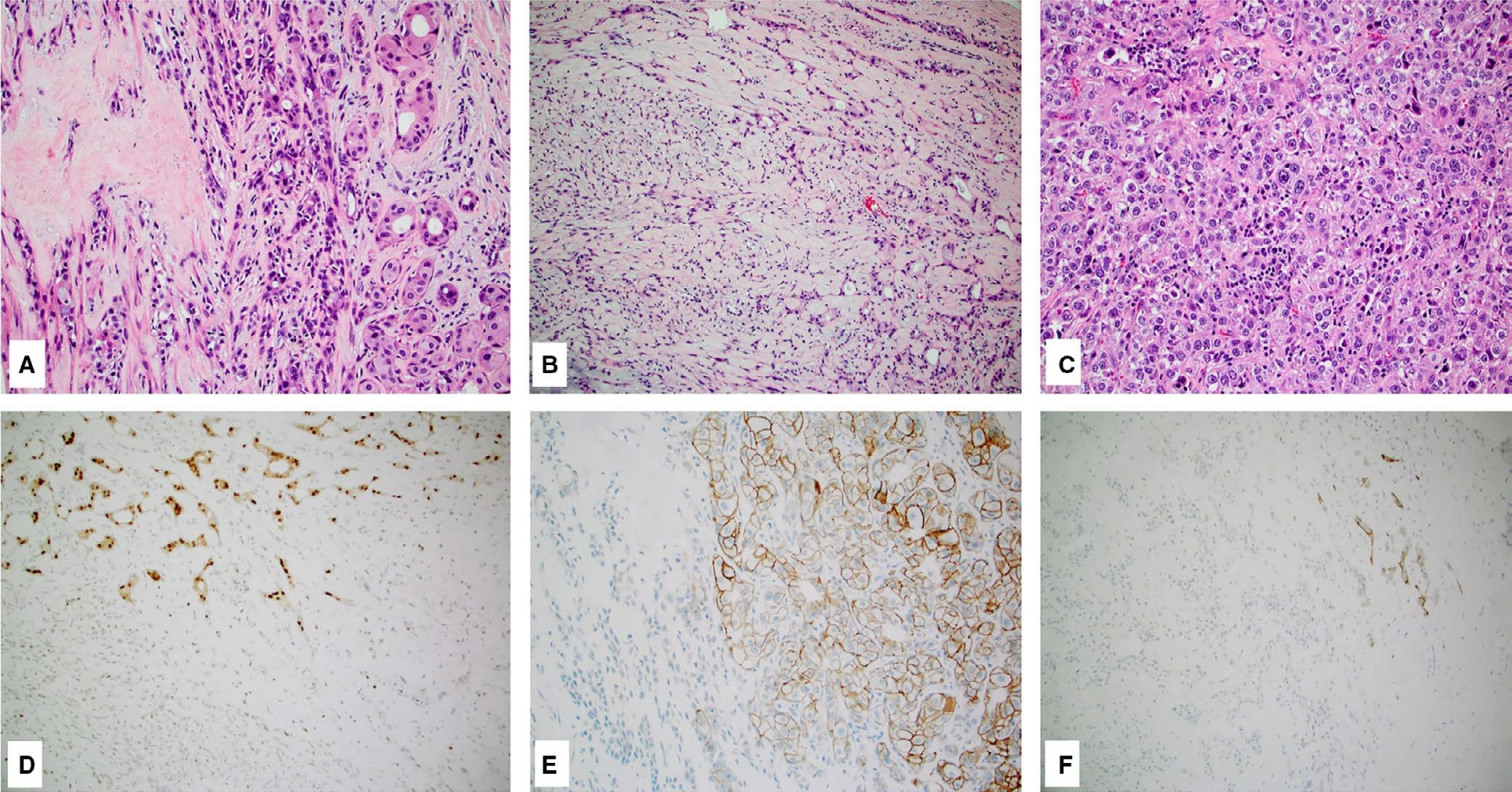
Case of salivary duct carcinoma ex pleomorphic adenoma (A‐C, hematoxylin and eosin, 10×) showing expression of androgen receptor (D, 10×), HER2 (E, 10×), and programmed death ligand 1 (F, 10×) restricted to the malignant component only

**Figure 2:**
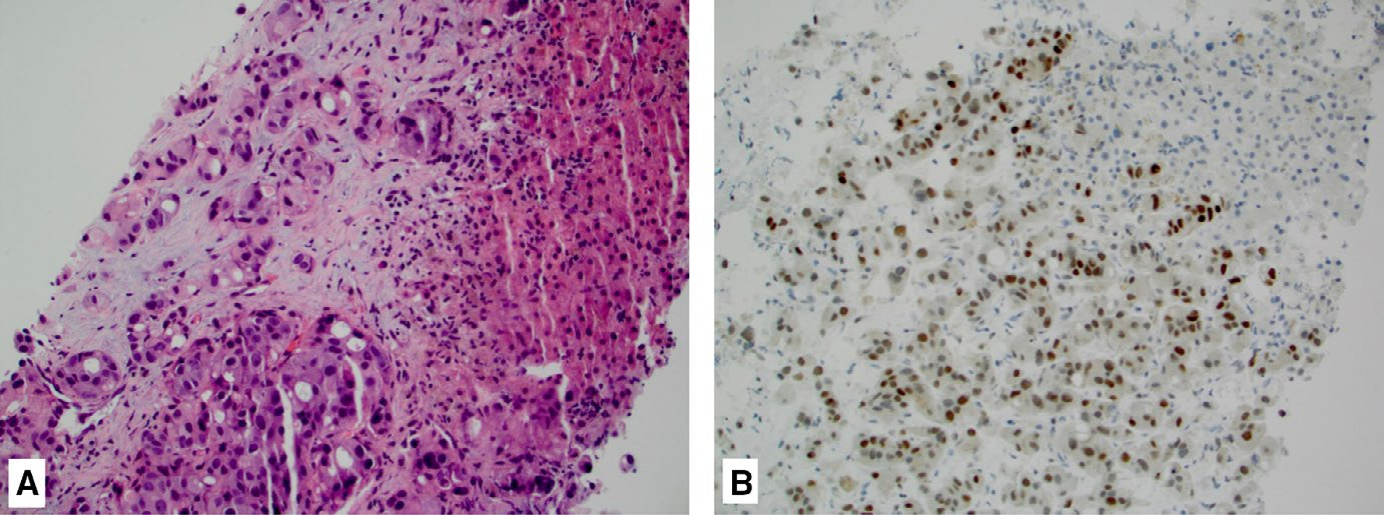
Case of metastatic salivary duct carcinoma in the liver (A, hematoxylin and eosin, 20×) showing diffuse nuclear expression of androgen receptor splice variant‐7 (B, 20×)

Results of molecular profiling assays are summarized in Table 2. HER2 was overexpressed in 39% (11/28) of the cases. All 28 cases expressed strong nuclear staining for AR by IHC (100%) (Figure 1D), and 13/27 (48%) harbored splice variant AR‐V7 detected by either variant‐specific antibody (showing nuclear localization) (Figure 2) or mRNA (Table 2). AR‐V7 positivity by IHC (n = 16) ranged from 10%‐95% (mean 53%) of tumor cells. A single case without any detected pathogenic mutation in our cohort had AR‐V7 present in the tumor.

**Table 2:** Summary of molecular genetic features of salivary duct carcinomas

Molecular genetic features		
Androgen receptor (AR)	100% positive (28/28) via IHC	13/27 (48%) had AR‐V7 (9 primary, 4 recurrent/metastatic)
HER‐2/neu receptor	11/28 (39%) positive via IHC	
Mutational profile^a^	n = 19 (68%): *TP53* n = 7 (25%): *HRAS* n = 5 (18%): *PIK3CA*, *NF1* n = 3 (11%): *MLL3*, *PTEN* n = 2 (7%): *BRAF*, *ERBB2*, *KDM5C*, *NOTCH1* n = 1 (4%): *FGFR1*, *CDKN1B*, *CREBBP*, *MLL2*, *CHEK2*, *RB1*, *BAP1*, *AKT1*, *PBRM1*, *SF3B1*, *ARID1A*, *SMARCE1*, *KMT2C*, *FBXW7*, *TSC2*, *PIK3R1*, *ATRX*, *C176Y*	
Gene amplifications	*NOTCH1* (n = 2, both primary) *ERBB2* (n = 4, 3 primary cases and 1 metastatic case) *SDC4* (n = 1, primary case) *EGFR* (n = 1, metastatic case) *MDM2*, *LGR5*, *KDM5A*, *ERC1*, *CDC73* (all in one metastatic case)	
Archer fusion panel (n = 12)	*WASF2*:*FGR* (n = 1) *TBL1XR1*:*PIK3CA* (n = 1) *FGFR2*:*PAWR* (n = 1)	

Predictors to immune checkpoint inhibitors		
PD‐L1 expression^b^	23/28 (82%) negative 5 cases (18%) with low PD‐L1 positivity (1%‐8% positive cancer cells)^b^	One case with *PD‐L1* amplification
Tumor mutational burden (TMB)	Low (range 3‐6) in 20/22 (91%) High (14 and 16 mutations per Mb) in 2/22 (9%)	
Microsatellite instability status	100% (18/18) microsatellite stable	

Abbreviations: AR‐V7, androgen receptor splice variant‐7; IHC, immunohistochemistry; PD‐L1, programmed death ligand 1.

a Full sequencing was performed using two different platforms: the Caris Life Sciences (n = 22) and Foundation Medicine (n = 6).

b PD‐L1 expression was assessed by 28‐8 (Agilent) and SP142 (Ventana) clones.

A case with matched primary and metastatic SDC harbored identical *TP53* and *ERBB2* mutations in both samples. A case of SDC ex PA harbored a *TP53* mutation in the carcinoma component, while the PA component was devoid of any mutation. Additionally, in this case (Figure 1A‐C), HER2 was positive in the carcinoma but negative in the adenoma component (Figure 1E). Both PA and SDC were devoid of AR‐V7.

### 3.4 Genomic profile of SDCS

Pathogenic genetic alterations were detected in 27 of 28 cases, many of which harbored more than one alteration. The most frequently encountered were *TP53* (n = 19), *HRAS* (n = 7), *PIK3CA* (n = 5), *NF1* (n = 5), *ERBB2* (n = 5) (3 cases with amplification alone, 1 [matched primary and metastatic case] with point mutation p.L755S alone, and 1 case with amplification and two point mutations: p.R678Q, p.V842I), *KMT2C* (n = 3), *PTEN* (n = 3), *BRAF (*p.V600E)*, KDM5C* and *NOTCH1* (n = 2 each). All other mutations were rare and affected single SDCs (Table 2). Co‐mutations of *HRAS* (p.Q61R) and *PIK3CA* (p. E542A, p.H1047R) genes were seen in 5 of 7 cases with *HRAS* mutations, all of which were de novo SDC with concomitant HER2 overexpression.

Gene fusion results, available for 12 cases from Caris, were rare and included *WASF2*:*FGR, TBL1XR1*:*PIK3CA,* and *FGFR2*:*PAWR* fusions in single cases (Table 2).

### 3.5 I‐O biomarkers in SDCS

Five cases had detectable (≥1% of tumor cells) PD‐L1 expression, and only 2 cases exhibited more than 5% tumor cell PD‐L1 expression. One case showed *PD‐L1/JAK2* gene co‐amplification by NGS but was negative for PD‐L1 expression by IHC. Immune cells expression of PD‐L1 was detected in 5 cases (3 cases without detectable TC PD‐L1 expression) but was not scored due to the lack of uniform criteria.

All tested samples (n = 18) were microsatellite stable (MSS). Twenty of 22 tested cases had a low TMB (range 3‐6 mutations/Mb), while 2 cases (one primary and one recurrent SDC) had TMB > 10 per Mb (14 and 16 mutations per Mb). Both of these cases were PD‐L1 negative.

## 4 DISCUSSION

In this study, we explored the molecular genetic characteristics of a well‐defined cohort of patients with SDC. Our study revealed common mutations affecting the mitogen‐activated protein kinase (MAPK) and PIK3CA/AKT pathways along with consistent AR and frequent HER2 expression. The molecular genetic landscape of our SDC specimens is comparable to findings in other studies on SDC and closely resembles that of apocrine breast cancer.^2^, ^29^, ^30^

Half of the tumors studied harbored alterations in MAPK pathway (*HRAS/BRAF/NF1*) genes, which is in line with a recent study of Dalin et al^30^ *HRAS* gene mutations were particularly common (25%) indicating their significant oncogenic potential in SDCs. These mutations were not mutually exclusive with *PIK3CA* mutations as co‐mutations were present in 5 of 7 *HRAS‐*mutated cases. This observation was recently reported in SDC by Dalin et al^30^ and previously in other cancer types.^31^ In contrast to Dalin et al,^30^ who found co‐occurring *PIK3CA* mutations in all of their cases with *HRAS* p.G13R (n = 3) but none of their cases with *HRAS* p.Q61R (n = 4), all 5 of our *PIK3CA/HRAS* co‐mutated cases harbored the *HRAS* p.Q61R mutation. Therefore, both *HRAS* p.Q61R and p.G13R seem to interact with *PIK3CA* in promoting oncogenesis in SDC. Furthermore, combined *HRAS/PIK3CA* mutations were found in de novo SDC cases and none of our SDC ex PA cases, which is a similar finding to that of Chiosea et al.^32^ The finding of frequent *PIK3CA/HRAS* co‐mutations in SDC may be clinically relevant as a potential cause of anti‐HER2 resistance given that all 4 of our cases with *PIK3CA/HRAS* co‐mutations were also HER2‐positive. However, none of the 12 patients from our cohort has been treated with anti‐HER2 agents although previous data indicate a significant clinical benefit of trastuzumab‐based therapy among patients with HER2‐positive SDC.^6^, ^30^, ^33^, ^34^ More recent studies suggest that dual HER2 inhibition with trastuzumab plus pertuzumab, which is a HER2 dimerization inhibitor antibody, or trastuzumab plus chemotherapy, may lead to even better clinical outcomes when compared to trastuzumab alone.^35^, ^36^

While mutations in *PIK3CA*, *HRAS,* and *BRAF* seem to be more common in de novo SDC, SDC ex PA have been reported to more frequently overexpress HER family members such as HER2, HER3 (ERBB3), and EGFR.^37^ In our study, 4 of 5 SDC ex PA, 5 of 13 de novo SDC, and 2 of 10 recurrent/metastatic SDC were positive by HER2 IHC. In a large series of 151 SDCs, *PIK3CA/HRAS/BRAF* mutations and HER2 positivity were reported to be mutually exclusive.^37^ In contrast, in our 7 cases with *PIK3CA/HRAS/BRAF* mutations, 4 cases (all with *PIK3CA/HRAS* co‐mutations) showed HER2 expression by IHC. However, the 2 cases with *HRAS* mutation alone, along with 1 case with *BRAF* p.V600E, were negative for HER2 IHC. Additionally, the 2 cases with *HER2* amplification did not show *PIK3CA/HRAS/BRAF* mutations, suggesting that perhaps *HER2* amplification but not HER2 overexpression by IHC alone may be mutually exclusive with these mutations.

The presence of *PIK3CA* mutations in a subset of SDC represents a viable therapeutic target. A large series of patients with various tumor histologies showed that the presence of the *PIK3CA* p.H1047R mutation specifically was an independent predictor for tumor response to PI3K pathway inhibitors.^38^ All 4 of our patients with *PIK3CA* mutations had this particular mutation, and one had a concurrent *PIK3CA* p.E542A mutation. Besides *PIK3CA* mutations, loss of *PTEN* activates the PI3K pathway and thus may represent another potential target. Several studies have reported frequent loss of PTEN expression, detected via IHC or FISH analysis, in up to 51% of SDC.^15^, ^37^ In head and neck squamous cell carcinoma, tumor cells with PTEN loss were shown to be commonly resistant to pan‐PI3K inhibitors; however, in vitro studies have shown promising results for targeted PI3K therapy in SDC cells.^15^ Finally, the discovery of a *TBL1XR1‐PIK3CA* fusion, which has been reported to result in overexpression of the complete PIK3CA protein, in one of our cases further supports the important role of *PIK3CA* in the oncogenesis of SDC.^39^

The *BRAF* p.V600E mutation was detected in two of our cases, similarly to the previously reported studies.^2^, ^32^, ^37^ Although the overall percentage of SDCs with mutated *BRAF* may be low, there is a potential for targeted therapy for these patients, as *BRAF* inhibitors are used successfully in melanoma and other *BRAF*‐mutated tumors. A phase 2 study of vemurafenib in a variety of *BRAF* p.V600 mutation‐positive non‐melanoma cancers showed partial response in 1 patient with SDC.^40^ Additionally, a recent report describes a patient who presented with widely metastatic disease and achieved an excellent response to combination chemotherapy with dabrafenib (*BRAF* inhibitor) and trametinib (*MEK* inhibitor).^41^ Clearly, routine testing for *BRAF* mutations in SDC seems warranted to select patients for targeted therapy.

The presence of AR‐V7, a biomarker predictive of resistance to anti‐androgen therapy in prostate carcinoma, in 13/27 (48%) of cases of SDC indicates a potential role for refining patient selection for hormonal therapies. The AR‐V7 splice variant encodes a truncated AR protein that possesses only the transactivating N‐terminal domain without the C‐terminal ligand‐binding domain (which normally serves as the binding site for anti‐androgen agents), resulting in constitutive activation of AR.^3^, ^8^ With the use of RT‐PCR or RNA sequencing, AR‐V7 mRNA has been reported in up to 50% of SDC tumors.^17^ Other AR isoforms such as AR‐V3 and AR‐45 are found less frequently and with unknown clinical significance.^8^ The presence of AR‐V7 in prostate cancer tumor cells has been associated with resistance to ADT, though there are limited studies thus far regarding its effect in SDC response to anti‐androgen therapy.^17^, ^42^ Cappelletti et al^16^ report a single patient with SDC treated with ADT who developed resistance and was found to have AR‐V7 in circulating tumor cells. In our study, the 2 patients treated with ADT did not have AR‐V7 in their initial tumor samples, and they showed clinical response in the form of stabilization of the disease. Interestingly, the tumors with AR‐V7 in our study harbored this protein prior to initiation of any systemic therapies, as the tissue tested came from tumor samples from the initial surgical resection. Therefore, it seems that prior exposure to ADT is not necessary for the development of AR‐V7 in SDC. Kang et al^17^ report a similar finding, in that SDC specimens from 2 patients with no prior exposure to ADT demonstrated high signal for AR‐V7 with RNA in situ hybridization (RISH). Additionally, they found that only 1 patient of 3 treated with ADT had clinical benefit, and this patient was AR‐V7 negative by RISH.^17^

We also explored the previously established predictive biomarkers to immune checkpoint inhibitors including PD‐L1, TMB and MSI status. Five of our cases exhibited low level (1%‐8%) PD‐L1 expression in tumor cells. A recent study by Sato et al,^43^ which utilized the PD‐L1 clone E1L3N (Cell Signaling Technology), revealed higher rates of PD‐L1 expression in SDC tumor cells, with 4 of 18 cases showing high expression (defined as greater than 10%) and 5 of 18 cases showing low expression (1%‐9%); moreover high PD‐L1 expression strongly correlated with shorter overall survival. In our study, two different anti‐PD‐L1 assays (laboratory developed SP142 assay and 28‐8 complementary diagnostic assay used for head and neck squamous cell carcinoma) were used in 2 different groups of cases, respectively. We found 1/12 TC positive using 28‐8 and 4/16 TC positive using SP142 clones. Importantly, all tested cases were MSI stable (tissue site agnostic assay for the use of pembrolizumab), while the vast majority (20/22) exhibited low TMB. Based on the obtained data for predictive biomarkers, SDC patients are less likely to benefit from the immune checkpoint inhibitors. In a clinical trial of 26 patients with PD‐L1‐positive advanced salivary gland cancer of various histologies, the objective response rate for pembrolizumab monotherapy was only 12%.^22^ However, additional trials (NCT03360890) are underway with the hope of achieving improved clinical response rates using combination pembrolizumab plus chemotherapy.

In summary, SDC is a genetically diverse neoplasm associated with several potentially targetable molecular pathways, indicting the need for individual patient's tumor assessment. Some of the detected molecular alterations (eg AR‐V7, *PIK3CA, HRAS*) may undermine the therapeutic benefits of standardized treatments (anti‐AR and anti‐HER2). The predictive biomarkers to immune checkpoint inhibitors indicate a small potential for therapeutic benefit of these agents in some patients.

## CONFLICT OF INTEREST

None declared.

## Data Availability

The data that support the findings of this study are available from the corresponding author upon reasonable request.
